# Supplementary material for: Eimeria tenella AMA1 regulates host cell apoptosis through the mitochondrial pathway and the death receptor pathway
Source: Microbiol Spectr. 2025 Aug 25;13(10):e00416-25. doi: 10.1128/spectrum.00416-25 (PMC12502623; doi:10.1128/spectrum.00416-25)
Supplement: Supplemental material — Fig. S1 and S2. [file spectrum.00416-25-s0001.doc]

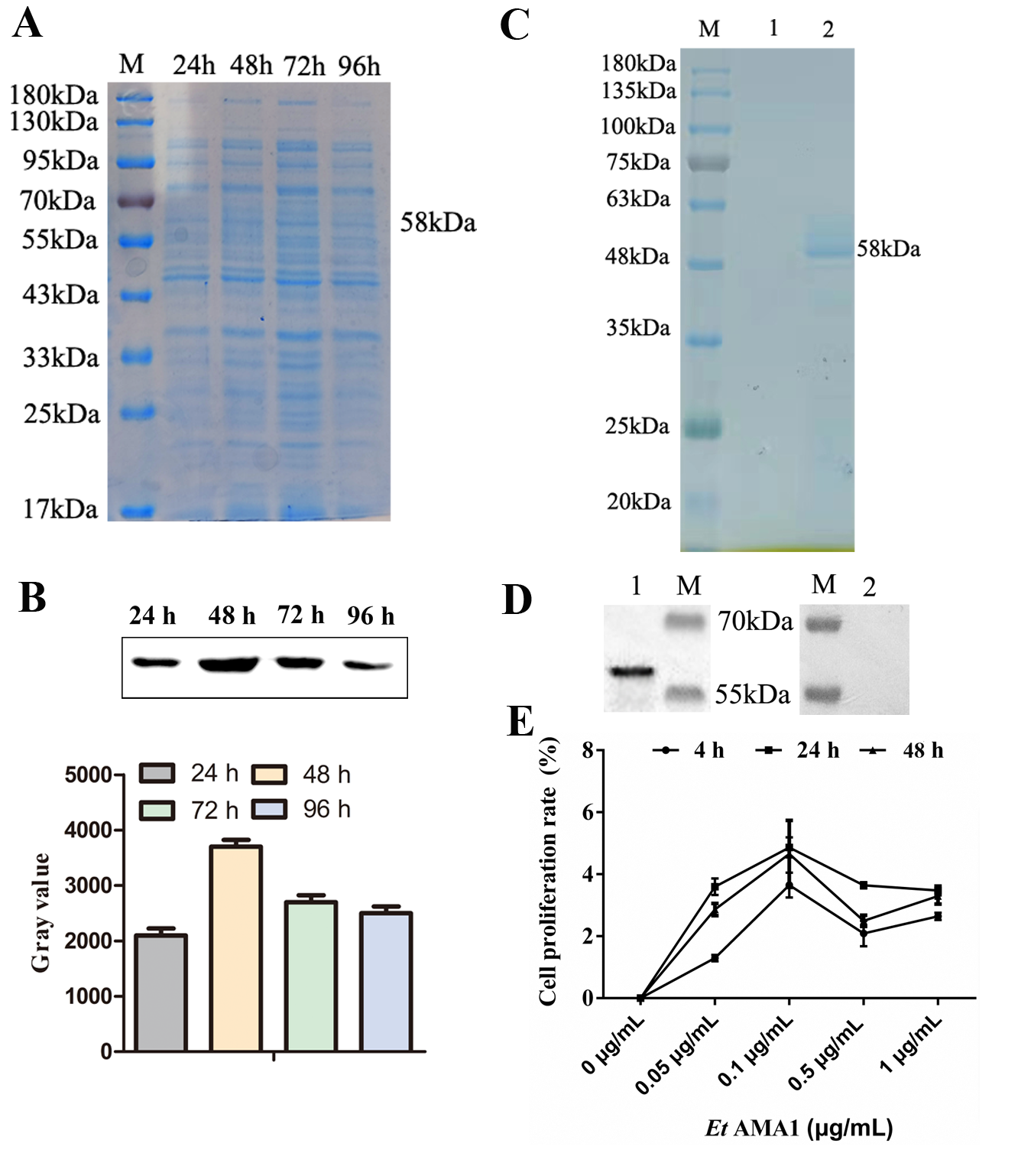


**Fig. S1. The construction of PPICZαA-*Et*AMA1 yeast expression system.**

1. Coomassie bright blue staining determined the induction expression time of *Et*AMA1. (B) *Et*AMA1 recombinant protein expression at each time period. (C) Coomassie bright blue dyeing. M: protein molecular weight standard; 1: negative control; 2: *Et*AMA1 recombinant protein. (D) Western blotting. M: Marker; 1: *Et*AMA1 recombinant protein; 2: negative control.


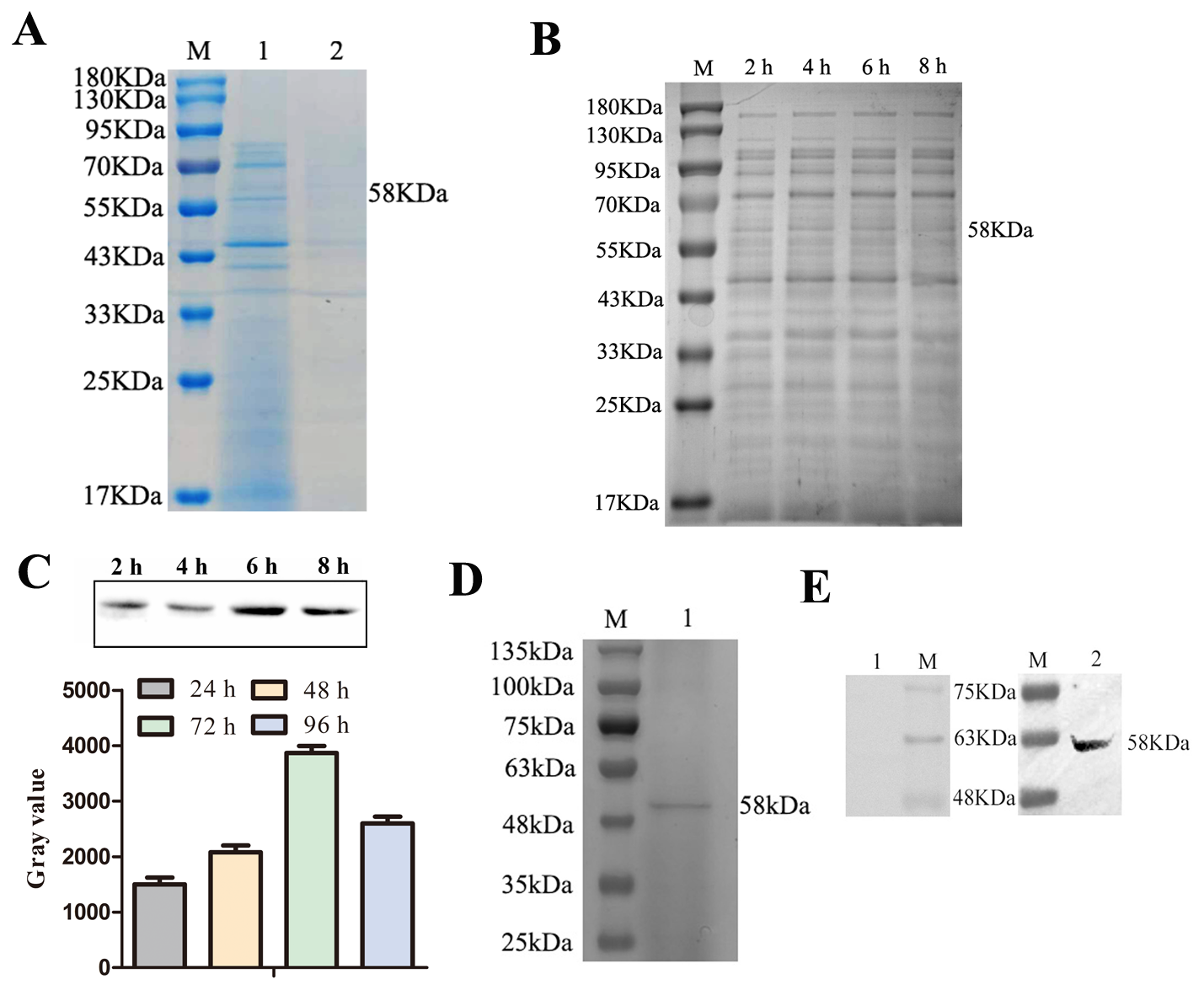


**Fig. S2. The construction of pET- *Et*AMA1 yeast expression system.**

1. Coomassie bright blue staining of *Et*AMA1 protein. M: Marker; 1: supernatant after ultrasound; 2: precipitation after ultrasound. (B) Determination of induction expression time of *Et*AMA1 protein. (C) *Et*AMA1 recombinant protein expression at each time period. (D) Coomassie brilliant blue staining; M: Maeker, 1: *Et*AMA1 recombinant protein. (E) Western blotting. M: Marker; 1: negative control; 2: *Et*AMA1 recombinant protein.
